# Complication-Free Intra-operative Mismatched Blood Transfusion Under General Anesthesia With an Interdisciplinary Approach: A Case Report

**DOI:** 10.7759/cureus.89128

**Published:** 2025-07-31

**Authors:** Ahella Jastanyah, Rahaf I Niyaz, Ahmed Bokhari, Mubashar Zia

**Affiliations:** 1 Anesthesiology, King Abdulaziz Medical City, Jeddah, Jeddah, SAU

**Keywords:** general anesthesia practice, hemolysis, incompatible blood group, intra-operative, mismatch transfusion

## Abstract

This report provides a case of a 58-year-old female scheduled for bilateral total knee arthroplasty under general anesthesia who received one unit of packed RBCs intraoperatively. She remained vitally stable throughout the whole procedure. However, by the end of the procedure, it was noticed that the administered blood was incompatible with the patient’s blood group. All patient-protective measures were taken including an ICU bed and hematology team consultation. Fortunately, no hemolysis or major complications emerged. The patient was discharged home in good condition by day 6 postoperatively.

## Introduction

Blood transfusion reactions can be divided roughly into infectious and non-infectious, immune-mediated and non-immune mediated, in addition to immediate and delayed reactions. When talking about immediate complications, the most serious one is intravascular hemolytic transfusion reaction due to ABO-incompatibility blood transfusion. In this case, we discussed an incident where a mismatched blood transfusion was done intra-operatively under general anesthesia. We can observe a gradual drop in hemoglobin levels and elevated lactate dehydrogenase (LDH) levels during the admission days post-operatively, yet there were no significant signs of hemolysis either clinically or laboratory-wise. It is important to note that the presence of general anesthesia may mask the symptoms of hemolytic and nonhemolytic transfusion reactions, such as hypotension, tachycardia, and hematuria. However, in this case, none was present [1,2].

## Case presentation

A 58-year-old diabetic and dyslipidemia female patient was admitted for elective bilateral total knee arthroplasty under general anesthesia. Patient demographic data is provided in Table 1. Pre-operative assessment and history were taken, and laboratory results were reviewed to be within acceptable limits for the procedure. Blood typing and cross-matching showed that the patient’s blood type is O+. Informed consent for anesthesia, surgery, and blood transfusion was obtained and signed by the patient before entering the operating room.

**Table 1:** Patient demographics

Characteristics	
Age	58
Gender	Female
Past medical history	Diabetes mellitus on metformin dyslipidemia
Weight	57kg
Height	151cm

The patient was pre-medicated with 2mg midazolam in the holding area and then pushed into the operation theatre. Standard American Society of Anesthesiologists (ASA) monitors were attached, and all parameters were within normal. Spinal anesthesia was administered by the consultant, using an aseptic technique. Local anesthetic was injected first, then the spinal anesthesia was injected by pencil point needle. Hence, induction of anesthesia was initiated. The patient received 150mg of propofol, a total of 100mcg of fentanyl, 40mg of rocuronium, and a prophylactic dose of antibiotic. Then, a supraglottic airway was inserted for ventilation.

The patient was vitally stable following the induction of anesthesia. A urinary Foley catheter was inserted afterward. During the surgery, the patient was vitally reassuring throughout the replacement of the first knee. Under the surgeon's request, one unit of packed RBCs was to be transfused as it is a regular part of his practice during bilateral total knee replacements. The bag was transferred by the nurses to the OR floor fridge, and brought by the anesthesia technologist to the OR. The anesthesia team checked the information on the bag with a copy of the request form submitted to the blood bank, which was completely accurate. However, this was done without verifying it with the patient’s ID band or the information in the anesthesia record sheet. Blood transfusion starting record was signed by the consultant and administration of the blood was initiated. The patient remained stable after blood administration and was successfully extubated fully awake with no distress or complications. By the end of the procedure, it was discovered that the patient had received the wrong transfusion as the Medical Record Numbers on the bag and request paper were not identical to the patient ID band.

Both the Floor Manager and the Blood Bank were immediately informed. Samples for blood tests including complete blood count (CBC), liver profile, coagulation profile, and fibrinogen level were sent, in addition to urine samples for urinalysis and urine creatinine levels. Precautionary measures were also taken, including a 100 mL/hour infusion of lactated Ringer’s solution and the application of a 24-hour oxygen mask set to 2 L. An ICU bed was arranged, and the incident was reported before transferring the patient for thorough monitoring over the first 24 hours.

Breaking the bad news of the mismatched transfusion was done using a multidisciplinary approach, consisting of the anesthesia consultant, orthopedic consultant, hematology consultant, nursing manager, and the patient experience staff. The patient received the news well, and her concerns were all answered.

In the first 24 hours, the patient remained hemodynamically stable. CBC differentials, liver profile, fibrinogen, urinalysis, and urinary creatinine were run every few hours. It is also important to note that repetition of blood typing and cross-matching was done to exclude an error in the pre-operative blood group result. She was then discharged from the ICU to the ward in good condition.

**Figure 1:**
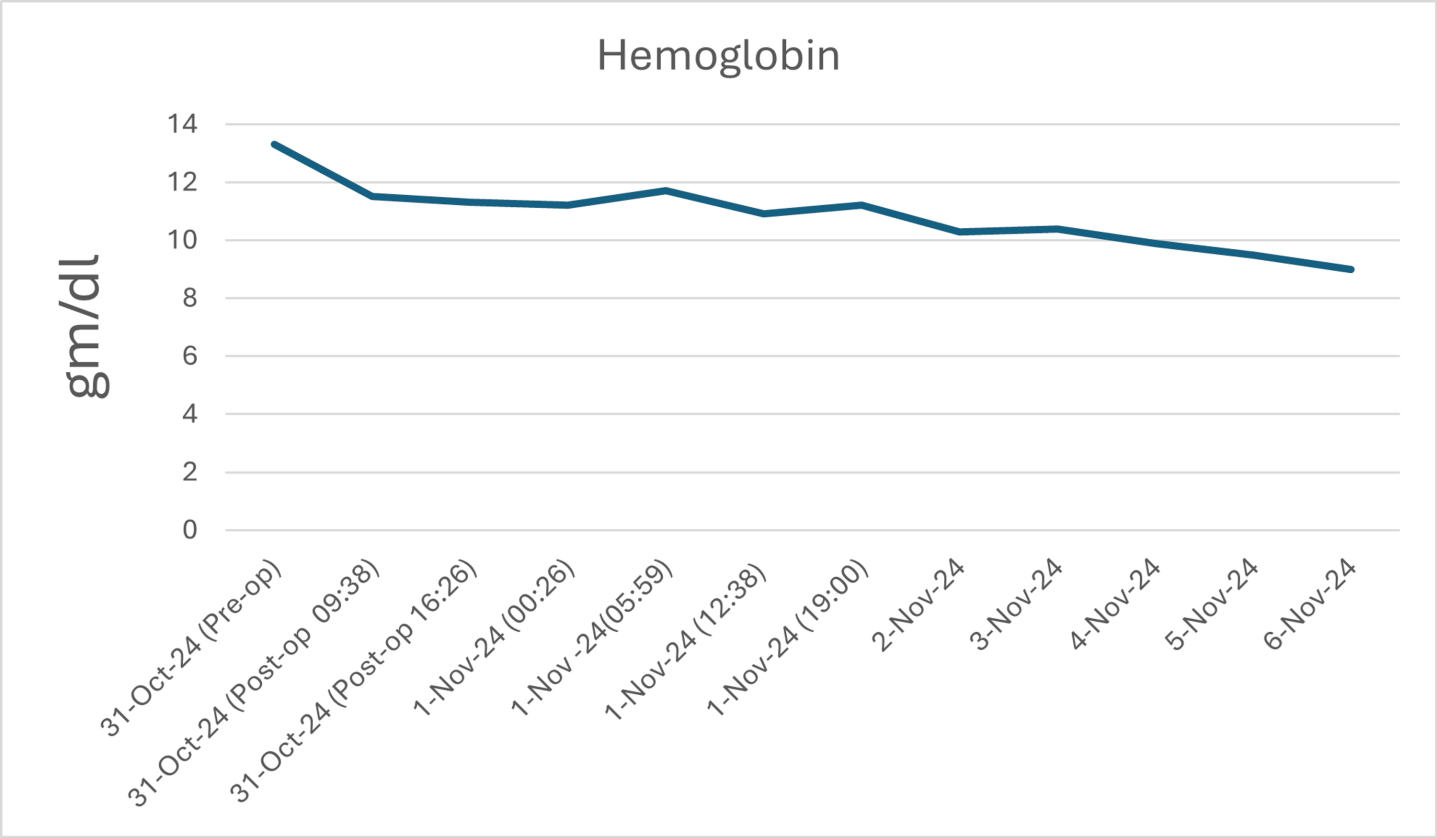
Hemoglogbin levels follow-up

**Figure 2:**
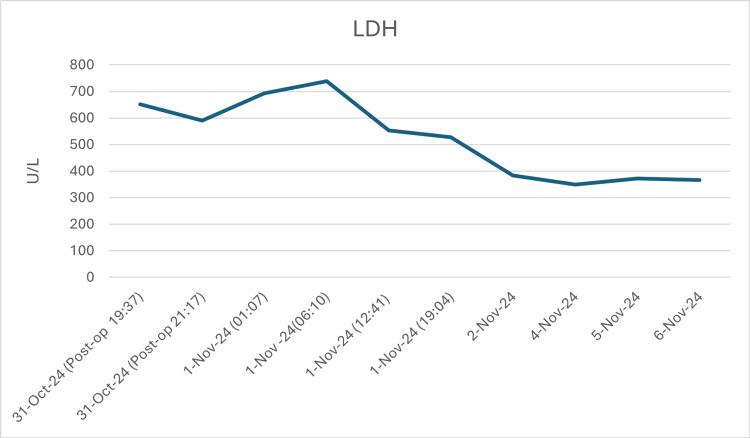
Lactate dehydrogenase (LDH) levels follow-up

**Figure 3:**
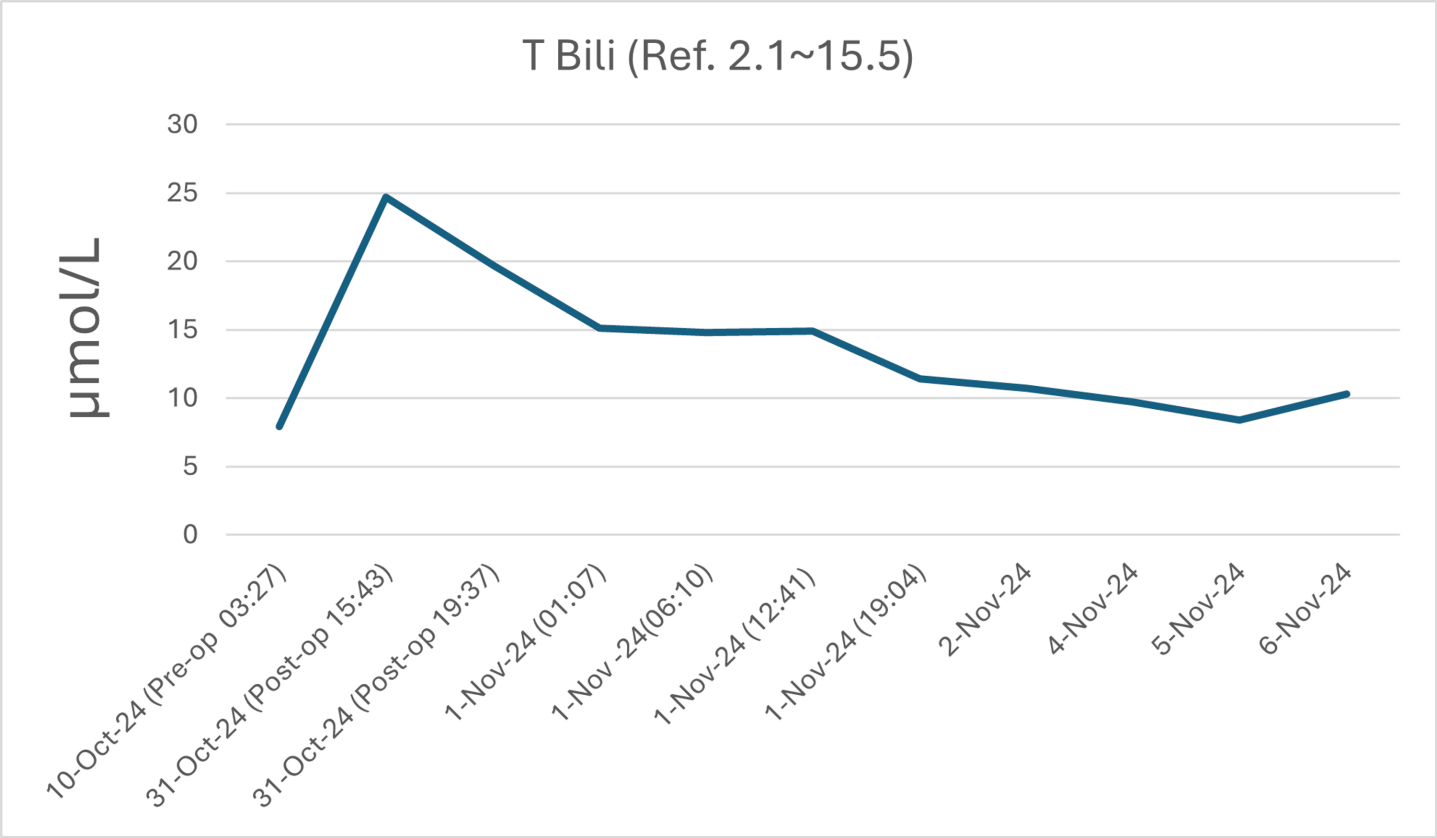
Total bilirubin levels follow-up

During the days of her admission, the patient was closely monitored for hemolysis by the hematology team and the primary orthopedic team. Several investigations were advised to be done daily by the hematology team, including the Coombs test, haptoglobin, LDH, total and indirect bilirubin, and urinalysis. The patient was vitally stable during the admission with no clinical evidence of hemolysis or hypersensitivity reactions. Moreover, no hemolysis was observed lab-wise apart from a gradually dropping hemoglobin and a transiently high LDH as shown in Figures 1-3.

By the sixth day post-operatively, the patient was stable, mobilizing well, tolerating orally, passing flatus and urine normally, and not complaining of any manifestations of hemolysis or hypersensitivity. The primary orthopedic team decided to discharge the patient, and the Hematology team approved that the patient was fit for discharge after reviewing all her lab's pre- and post-operative as shown in Table 2. The patient was set for discharge with red flags of hemolysis given and an outpatient appointment in the hematology clinic after one week.

**Table 2:** Pre-operative, post-operative, and at discharge parameters

	Pre-operative	Post-operative Day 1	At Discharge	Unit
Hemoglobin	12.3	11.2	9	g/dL
Reticulocytes	45	45	62	x10^9/L
Total Bilirubin	7.9	15.1	10.3	umol/L
Direct Bilirubin	3.7	7	5.8	umol/L
Alanine Transferase (ALT)	24	24	-	U/L
Aspartate Aminotransferase (AST)	25	47	-	IU/L
Creatinine	6.7	3.8	-	mmol/L
Blood Urea Nitrogen (BUN)	6.1	5.6	-	mmol/L

## Discussion

This report presents a case of ABO-incompatible blood transfusion administered to a female patient, which fortunately concluded without any complications. The ASO provided practice guidelines for peri-operative blood transfusion and adjuvant therapies. It includes reviewing the patients’ precious records, as done in this case. In addition, it advises continuously monitoring vital signs, peak airway pressure, and urine color after administration of the blood. Of which all were implemented in our case, no change in patients’ hemodynamics, airway pressure, or urine color was documented [3,4].

A previous report from Turkey described another case of an intraoperative mismatched transfusion involving 1 unit of packed RBCs and 1 unit of fresh frozen plasma (FFP) during a pediatric cardiopulmonary bypass, which fortunately ended favorably. In contrast to this article, their anesthesia team discovered it intra-operatively due to hematuria. Blood was seen in the urine bag and proper management was then provided. They managed it immediately by intravenous pharmacological agents including methylprednisolone, heparin, potassium, furosemide, and sodium bicarbonate, followed by exchange transfusion. In our case, no immediate reaction was seen intra-operatively, therefore, the anesthesia team did not consider diuresis for the patient.

All parts of the “transfusion chain" are susceptible to errors, whether it is the laboratory personnel or the perfusionist, and it has been shown that human error and bedside misidentification are considered among the most prevalent ones. In our case, the leading cause of this accident was human error, in which the verification of the patient’s information and the received bag was not done, resulting in transfusing the wrong blood to the patient.

The previously mentioned article taking place in Turkey has proposed the need for constructing institutional protocols to prevent such hazards. Thus, after this incident, several measures were taken by the hospital to avert its recurrence. A grand meeting was arranged, in which all healthcare providers working in operation theaters were assembled. The event was disclosed to everyone contemporarily, ensuring the confidentiality of the patient and the staff involved. Then, instructions for the future were given. Closed-loop identification of the patient and the received blood was encouraged in which a hand-sized scanner is provided by the hospital, whereupon the barcodes available on the patient’s ID band and the blood bag are to be scanned and verified electronically in order to reduce the chance of human error [5-7].

It is a well-perceived point of weakness in this case where no clear evidence-based justification was provided by the surgeon regarding the indication for the blood transfusion, rather than that he had personally observed a better prognosis in patients receiving blood in contrast to those who didn’t. Yet, it is also important to take into consideration that all the measures taken, as well as the new protocols established in our institution afterwards the incidence, were all beneficial in terms of patient safety and hazard preclusion.

## Conclusions

In conclusion, multi-disciplinary, double-checking, and closed-loop communication while transfusing blood intra-operatively should be done to ensure the correct administration. The use of technology when accessible can facilitate the process with a high level of accuracy. Moreover, as observed in our case, adverse effects may or may not emerge following an ABO-incompatible transfusion. Yet, all measures and protocols must take place to guarantee patients’ safety and survival. Most importantly, it is crucial to outline and offer solutions to errors to prevent future hazards.
